# Experimental Study on SPR Array Sensing Chip Integrated with Microvalves

**DOI:** 10.3390/s24082540

**Published:** 2024-04-15

**Authors:** Wanwan Chen, Peng Wang, Bin Li

**Affiliations:** 1 Department of Precision Instrument, Tsinghua University, Beijing 100084, China; 2 Research Institute of Tsinghua, Pearl River Delta, Guangzhou 510530, China

**Keywords:** microfluidic system, pneumatic microvalve, electrolyte conductance, SPR array detection

## Abstract

This paper discusses a microfluidic system designed for surface plasmon resonance (SPR) sensing, incorporating integrated microvalves. This system is built from a layered structure of polydimethylsiloxane (PDMS) and polymethylmethacrylate (PMMA). The functionality of the microvalves is verified through a conductance method involving electrodes positioned at the microfluidic channels’ inlets and outlets. These microvalves can fully close at a control pressure of 0.3 MPa, with their operation depending on the duration of the applied pressure. The study further explores the coordinated operation of multiple microvalves to regulate the sequential flow of samples and reagents in the system. In SPR detection experiments, the microfluidic system is integrated with an SPR array sensing system to control the injection of NaCl solutions via the microvalves, and the observation of phase change curves in different chip regions are observed. The findings validate the microvalves’ dependability and suitability for use in SPR array sensing.

## 1. Introduction

A surface plasmon resonance (SPR) sensor is an optical sensor that detects changes in the refractive index on a metal surface to obtain information in biomolecular inter-actions occurring during biochemical reactions. This has the advantages of requiring no labeling, real-time performance, and high sensitivity, and it is thus an important tool in proteomics research [1,2,3]. Requirements for microfluidic systems for high-throughput SPR detection include the ability to accurately and efficiently deliver analytes and rea-gents while allowing various solutions to flow across the chip’s surface at the same speed. Microvalves are components that control the on-state and off-state of micro-channels, and optimally designed microvalves should be characterized by low leakage, low power consumption, fast response time, and linear operation capabilities [4,5,6,7].

Microvalves are usually fabricated based on piezoelectric, electrostatic, thermal, and pneumatic principles [8,9,10,11]. Pneumatic microvalves can be easily integrated with PDMS on the chip [12,13] and are widely used in SPR assays [14]. The microvalve is used to control the stability of the flow rate and arrange the injection of reagents and analytes, which directly affects the assay’s accuracy. Therefore, it is important to properly characterize the microvalves for the application of microfluidic systems.

Quake et al. used pneumatic microfluidic chips to realize the large-scale integration of thousands of microvalves and hundreds of reactors on microfluidic chips, with a density of thousands of microvalves per square centimeter, which became an important technological breakthrough in the field of microfluidic chips [15]. Araci et al. proposed the ultra-large-scale integration of pneumatic microfluidic chips with a density of millions of microvalves per square centimeter [16]. Lee et al. studied the relationship between the closing performance of pneumatic membrane valves and the shape of the flow channels [17]. In light of the progress of multi-layer soft lithography techniques, several studies have described fabrication methods for high-aspect-ratio fluidic [18,19,20,21,22]. In recent years, pneumatic microfluidic chips have been used with increasing success in biological and chemical analyses [23].

Currently, convenient methods used to test the characteristics of microvalves include observation and conductivity methods. Li et al. characterized the on/off states of a microvalve by observing the deflection of the valve membrane through a microscope [24], and Studer et al. also used a microscope to observe the interfaces of two solutions, indirectly characterizing the operating state of the studied microvalve [25]. These two methods provide intuitive information but are not quantitative. Galas et al. used the quantitative conductance method to study the opening and closing process of a single microvalve [26]. They set up electrodes that were deposited in microchannels through a complicated process, which could result in measurement errors. In addition, how to characterize multiple microvalves at the same time is also a problem.

The integration of microvalves in microfluidic systems is a necessary step towards automation and high-precision inspection. Pneumatic microvalves are integral pieces of the basic components of a microfluidic chip. In recent years, some scholars have studied the deformation performance of pneumatic PDMS-driven films at different pressures, but there are not many studies on the application of pneumatic microvalves in SPR array detection. Research on the overall detection performance of pneumatic microvalves, such as the response time and the closing characteristics, which are still not perfect, is especially scarce. This study designed a microvalve using finite-element simulation and then established a multilayer structure for the microfluidic system according to the requirements for SPR array detection using PDMS and PMMA as the primary materials. The conductivity detection device is designed to capture the dynamic characteristics of the microvalve by altering its conductivity during the opening and closing processes under different pneumatic pressures. Additionally, the microfluidic device was applied in SPR detection, where the microvalves automatically control the injection of sodium chloride solutions at varying concentrations. The results indicate that the designed microvalves are practical and reliable and are suitable for SPR array detection. The proposed method can also be used to manufacture various other high-density micro-chips.

## 2. Design and Fabrication of Microfluidic Systems

### 2.1. Structure of the SPR Array Detection System

The detection system includes a microfluidic feeding system, and an optical path system built in a black box surrounded by a metal plate. The upper part of the instrument houses the microfluidic sampling system, temperature control system, and mechanical control system. The microfluidic feeding system is composed of an industrial syringe pump, a sampling needle, a needle-washing tank, a microfluidic chip and a liquid pipeline. The chip compression frame is used to press the fluid cell of the microfluidic chip and the sensing chip together. Externally, there are also waste and buffer bottles placed on a stretching platform. All circuit boards are integrated together to enable centralized power supply and data acquisition, transmission, processing, and, finally control and display via user PC software.

### 2.2. Design of Microvalves

#### 2.2.1. Materials for Microfluidic Systems

The microfluidic system designed in this paper is based on SPR detection, which requires fluid pools with a certain area and an open surface that, in turn, must be sealed to avoid leakage onto the sensing chip. The complete microfluidic system is composed of a multilayer structure. Given that PDMS is a highly elastic polymer material that can achieve reversible sealing on smooth surfaces, it was selected as the base material for constructing the microfluidic system, specifically Dow Corning’s two-component Sylgard184. Both the base and the curing agent of the two-component system are flowable liquids, mixed at a weight or volume ratio of 10:1. After the mixture, it forms a flexible elastomer with minimal shrinkage, and the curing process does not release heat, solvents, or curing by-products. The elasticity of the cured PDMS is independent of the encapsulation thickness and the degree of environmental sealing.

However, using PDMS as a structural material for the microchannels could lead to their deformation and blockage when pressure is applied to achieve a reversible seal between the sensing chip and the fluid pool. Therefore, introducing another rigid substrate material was considered to provide structural support in addition to improving the rigidity of the pneumatic channels, which need to withstand the external air pressure and the creation of threaded holes for the connection to the external pneumatic fittings that need to be machined. Plexiglas, a transparent rigid polymer material, was selected as the substrate material. Moreover, PMMA is a hard, easy-to-process material that has a mature polishing process and good transparency after polishing, which makes observation easier, and it is therefore selected as the main material for the airway layer. In addition, cured PDMS can be easily demolded from PMMA, and it is also used to make positive molds for microchannels and microfluidic cells.

Additionally, the control layer and the auxiliary layer are connected using pressure-sensitive adhesive (Adhesives Research). The external liquid tubing is made of Teflon material, which the PDMS microchannels cannot directly connect to. The material should possess excellent mechanical properties, good self-lubrication, resistance to chemical corrosion, good sealing, and biocompatibility. Polyether ether ketone (PEEK) is a high-performance polymer material that meets all these requirements; hence, it was chosen as the material for the compression block. In summary, the materials selected for this design primarily include PDMS, PMMA, PEEK, and double-sided adhesive.

#### 2.2.2. Microvalve Structure Design

**Figure 1:**
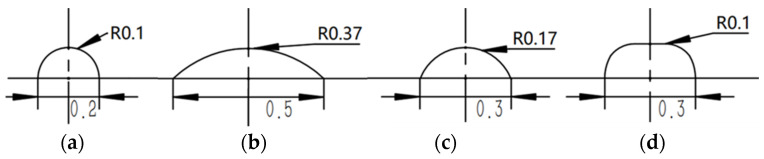
Cross-sectional diagram of the microchannel: (**a**) NO.1; (**b**) NO.2; (**c**) NO.3; (**d**) NO.4.

The characteristics of microvalves are largely determined by parameters such as the control pressure, the radius of the microvalve, and the width and height of the liquid channel, as well as the thickness of the membrane. If the membrane is too thick or too thin, it may be difficult to deform or be prone to rupture. The air pressure, the distance from the fixed end to the center, and the thickness of the membrane are all related. Moreover, whether a microvalve can completely shut off a channel also depends on the height of the channel. The wider the microchannel, the easier it is to achieve a complete shut-off. However, the wider the channel, the larger the size of the microfluidic system, and the greater the amount of sample consumed. Therefore, after testing, the width of the microchannel was preliminarily determined to be 200–500 μm. Obviously, the diameter of the microvalve must be greater than the width of the microchannel. With the same air pressure, the larger the diameter, the greater the shear force at the connection between the PDMS membrane and the airway layer, making it easier for the seal between the microvalve membrane and the airway layer to fail. Considering the manufacturing process, the diameter of the microvalve was chosen to be 1 mm. The thickness of the microvalve membrane is also an important design parameter. If it is too thick, deformation is difficult, requiring higher air pressure loads, increasing the difficulty of sealing the airway layer and microvalve membrane, and reducing the reliability; if it is too thin, it is prone to fatigue failure, making it difficult to recover its original elasticity or even causing rupture. Through experiments with PDMS membranes of different thicknesses, the thickness of the membrane was set to 100 μm. To facilitate manufacturing and better microvalve shut-off, the height of the microchannel was set to 100 μm. Based on the above parameters, the cross-sectional shape and size of the designed microchannel are shown in Figure 1.

**Figure 2:**
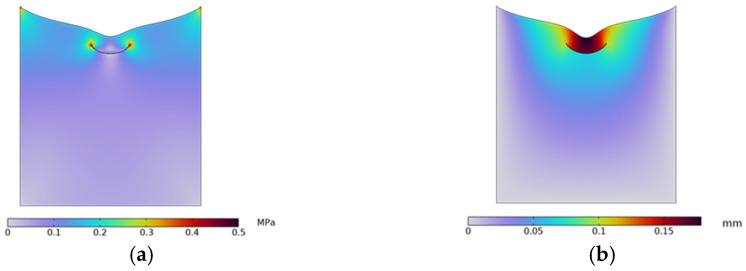
Simulation outcomes for stress and strain of microchannel NO.1: (**a**) stress distribution; (**b**) strain distribution.

To analyze the relationship between the closure of the microvalves, the channel shapes, and the applied loads, COMSOL 6.1 simulation software was employed to conduct finite-element analysis of the deformation of the microvalves within the different types of microchannels, as shown in Figure 2.

Taking design NO.1 as an example, when the load is P = 0.27 MPa, the deformation result is as shown in Figure 2a, and the microvalve is completely shut off. The maximum deformation is about 0.178 mm. The shear stress when the microvalve is completely shut off is shown in Figure 2b, with a maximum stress of about 0.46 MPa. The stress analysis at the connection points considers whether the seal between the microvalve membrane and the gas flow layer is reliable when the pressure load acts on the microvalve membrane, as well as the deformation and stress of the microvalve relative to other microchannels shapes. The cross-sectional area of four types of microchannels, the corresponding load pressure when completely shut off, and the maximum shear stress at the connection between the PDMS membrane and the upper gas flow layer are described in Table 1.

As can be seen from the table, when the microvalve is completely shut off and the air pressure and the maximum shear stress at the connection for the two types of microchannels are as shown in Figure 2; the designs NO.2 and NO.3 are both smaller, especially for NO.2, whose advantage is the most obvious. It can be seen that the arc-shaped channel is easier to shut off. The structures of the microchannels shown in NO.1 and NO.3 are smaller, which means they require smaller amounts of a sample or reagent, which is an advantage, with the design NO.1 using the smallest amount but being the most difficult to shut off. Considering both the achievement of shut-off and the amount of solution used, the design NO.3 is chosen as the microchannel design system for the microfluidic system.

For SPR array detection, the microvalve needs to control the entrance of the different solutions into the microfluidic pool in a timed manner. To this end, when designing the microchannel and the position of the microvalve, two issues need to be considered: First, the air in the microchannel must be expelled before the solution enters the microchannel. This requires the design of a bypass, so that the air in the channel can be expelled by the inflowing solution before it flows into the microfluidic pool, thus ensuring that the desired solution can immediately enter the microfluidic pool once the microvalve is opened. Second, after the microvalve is opened, the solution should be prevented from mixing with the previous solution as much as possible before it flows into the microfluidic pool. This requires that the path between the microvalve and the microfluidic pool be as short as possible, meaning the position of the microvalve should be as close to the microfluidic pool as possible. Compared to switch valve control sampling, microfluidic systems offer the following advantages:

(1) Within one experimental cycle, solution changeover can be accomplished by simply alternating the switching of Valves 1 and 3 and 2 and 4. By controlling the actuation pressure via a computer, simple and reliable sampling automation can be achieved.
(2) The mixing path of the buffer and sample solutions is just a short microchannel before entering the microfluidic pool, thereby preventing the mixing of sample solutions with the buffer before they enter the microfluidic pool and ensuring the accurate measurement of the kinetic constant.

### 2.3. Microfluidic System Assembly Procedure

In the designed SPR detection device, the microfluidic pool in the microfluidic system needs to be positioned above the chip’s surface, with solutions flowing into the microfluidic pool through the microchannels. This necessitates the arrangement of microchannels and the microfluidic pool on adjacent layers. Additionally, the direction of the fluid flow in the microchannels needs to be controlled by the adjacent microvalves, meaning the microchannels and microvalves also have to be placed on neighboring layers. Integrating the above analysis, the structural design of the microfluidic system is created. It features a multi-layered structure, comprising the air control layer, microvalve membrane layer, and fluidic layer. The control pressure acts on the microvalve membrane via the air control layer, causing it to deform and block the microchannel, thereby stopping the solution from entering the microfluidic pool. When performing detection tasks over a long period of time, large signal drifts can overwhelm the SPR response signal, thus requiring special attention. By combining the prism and the chip into one, the gold film can be plated directly onto the prism, constituting a one-piece sensing prism. In this way, signal drift due to the volatilization of refractive-index-matched oil can be eliminated, thus improving the stability of the system and facilitating continuous detection for a long period of time, as well as further eliminating changes in the refractive index caused by temperature fluctuations, which reduces the SPR signal drift.

**Figure 3:**
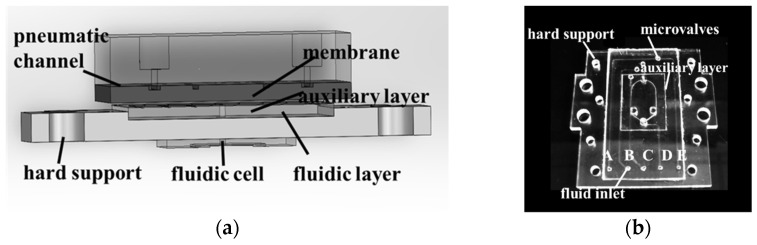
Microfluidic system assembly diagram: (**a**) diagram of partial mechanical assembly; (**b**) photograph of the actual object with the fluidic layer, microvalve membrane, and auxiliary layer sealed together as one entity.

After exploring and experimenting with various fabrication steps, CNC milling was preliminarily selected as the PMMA mold, molding techniques were used to process the PDMS microchannels, and the required through-holes at corresponding positions on the PDMS chip were cast during the molding process. Using oxygen plasma surface treatment, the obtained PDMS substrates were irreversibly bonded, and by employing a sandwich structure, a hard PMMA auxiliary layer for the SPR phase detection sensors was integrated into the overall structure of the microfluidic system, as shown in Figure 3.

The integration process of the entire microfluidic system is as follows:

(1) Two pieces of PMMA are sealed with AR pressure-sensitive adhesive to form the air channel and then set aside for 18 h before use;
(2) Using the oxygen plasma bonding method, the PDMS membrane on the Plexiglass plate is bonded with the auxiliary layer using oxygen plasma, and then demolded from the Plexiglass plate;
(3) Using a punch, holes are created corresponding to the auxiliary layer’s inlet on the membrane;
(4) Similar to Step 2, the other side of the membrane is bonded to the fluidic layer using oxygen plasma, but the microvalve positions need to be aligned during bonding;
(5) After plasma treating the other side of the auxiliary layer, AR double-sided adhesive is applied. Then, the air channel layer and the other side of the double-sided adhesive undergo oxygen plasma bonding and are set aside for 18 h;
(6) A PEEK compression block is pressed directly onto the auxiliary layer of PDMS substrate. After aligning their inlet holes, the PEEK compression block is fixed to the PMMA support with screw connections, resulting in a complete microfluidic system.

A comprehensive photograph of the actual microfluidic system is shown in Figure 3b. To further determine the actual conditions of the microchannels and microvalves, this study employed a white light interferometer to measure the cross-section of the microchannel mold. The measurement results show that the height of the microchannel was 114.2 μm, and the thickness of the microvalve membrane was 107.3 μm, both slightly exceeding the design values. This discrepancy is primarily attributed to errors in the molding process.

## 3. Microvalve Performance Testing

**Figure 4:**
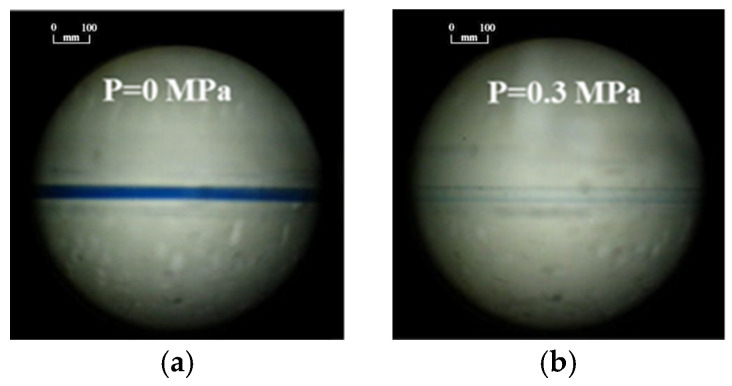
Micrographs of microvalve-controlled microchannels at different pressures: (**a**) P = 0 MPa; (**b**) P = 0.3 MPa.

To position the microfluidic system such that the microvalve is within the field of view, methylene blue solution is injected into the microchannel. Images captured without pressure on the microvalve and with a pressure of 0.3 MPa applied show a change from blue to no color beneath the microvalve, indicating the microvalve’s influence on the flow of the solution (Figure 4). However, this observational method cannot accurately determine the microvalve’s opening and closing times or its response frequency. Moreover, due to the limited field of view of the microscope, it is not possible to simultaneously monitor multiple valves, which is a requirement for SPR detection.

### 3.1. Principle of Conductivity Detection

**Figure 5:**
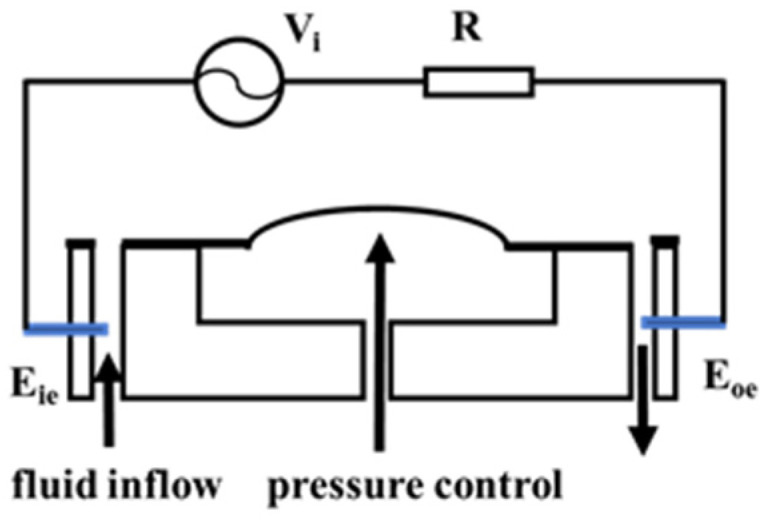
Schematic diagram illustrating the principle of conductivity method for microvalve characterization.

The conductance method is known for its ease of operation and intuitive results. Its detection principle is as follows: microelectrodes are etched into the channel, and when the microvalve deforms, the conductivity of the solution in the microchannel changes. This change in conductivity can be observed through variations in the current between the electrodes, thereby providing dynamic characteristics of the microvalve’s opening and closing actions. Based on this principle, the detection scheme is as shown in Figure 5. As illustrated, the input electrode E_ie_, the microchannel, the output electrode E_oe_, the external resistor R, and the voltage source Vi form a closed circuit. By measuring the change in voltage across resistor R, the dynamic characteristics of the microvalve can be understood. Before detection, a standard electrolyte solution is injected into the microchannel via a syringe pump.

This setup allows for precise and direct measurement of the microvalve’s functionality by translating mechanical deformations into electrical signals, offering an effective way to assess microvalve performance in real-time.

The value of the AC voltage output from the waveform generator is *V_i_*, the resistance of the measurement resistor is *R*, and the equivalent resistance of the liquid flow path of the thin-film microvalve is $R_{flu}$:

(1) $$R_{flu}=\kappa \times \frac{L}{S}$$

The value of the voltage *V*_0_ at the ends of the detection resistor is

(2) $$V_{0}=\frac{V_{i}}{R+R_{flu}}\times R$$

where κ is the conductivity of the solution, *S* is the cross-sectional area of the microchannel; and *L* is the length of the microfluidic channel to be measured.

When pressure is applied to the microvalve, the elastic membrane deforms, reducing the cross-sectional area of the microchannel, which in turn increases the equivalent resistance of the solution in the microchannel. By detecting changes in the voltage across resistor *R*, the deformation of the microvalve can be determined. If the microvalve is fully closed, the microchannel is completely blocked, making the equivalent resistance of the electrolyte solution in the channel effectively infinite, and the voltage across *R* drops to 0. Similarly, during the opening of the microvalve, *V*_0_ starts at 0 and gradually increases until the microvalve is fully open, at which point *V*_0_ reaches its maximum value.

This approach provides a straightforward and effective method of monitoring the operational state of the microvalve. It allows for real-time feedback on the valve’s status, which is crucial for systems requiring precise fluidic manipulation, such as in chemical synthesis, biological assays, or in lab-on-a-chip devices where the dynamic regulation of fluid flow is essential to the system’s functionality.

### 3.2. Conductivity Measurement System

**Figure 6:**
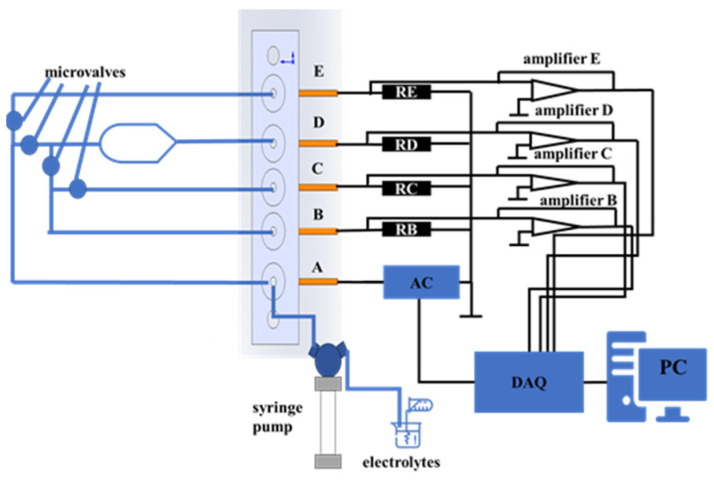
Schematic diagram of the microvalve detection experiment setup.

The experimental setup for microvalve testing, as shown in Figure 6, consists of a nitrogen gas source, a solenoid valve, the microfluidic system, an injection pump, a detection circuit, a data acquisition card (DAQ), and a computer. The signal processing setup mainly includes signal output, data collection, and display. Electrode A is connected to an AC voltage source, and Electrodes B–E are each connected to the external resistors RB-RE, which output and amplify the signal changes in the solution. Each path forms a closed circuit with the microchannel, Electrode A, and the voltage source. When the conductivity of the solution changes, the voltage source signal and the four output signals are collected by the DAQ and processed and displayed by the computer. To avoid the electrolytic effect of the DC voltage, the detection circuit’s power supply is a square wave with an amplitude of −2.5 V to 2.5 V and a frequency of 1 kHz. Depending on the needs of the microvalve detection task, this voltage source can be connected to either Electrode A or B. Before the experiment, the power source is connected to Electrode A. The choice of external resistors should consider the equivalent resistance value of the solution in the microchannel, and a standard 0.1 M potassium chloride solution is used as the electrolyte, injected into the microchannel by the injection pump. In this testing setup, the pressure acting on the microvalve is adjusted by the pressure valve on the nitrogen source. Thus, the opening/closing of the microvalve, controlled by the solenoid valve, is essentially computer-controlled, allowing for precise timing and execution.

At the start of the experiment, all microvalves are in the open state, and the electrolyte solution is injected from Inlet A, filling all microchannels and then flowing out from Outlets B–E. Then, the computer adjusts the solenoid valve to direct nitrogen gas into the airway connected to the microvalve being tested, causing the microvalve to deform. This deformation leads to changes in the flow state of the solution and its corresponding resistance, thereby changing the voltage across the external resistors. This change, output by the amplifier, is captured by the DAQ and then processed and displayed by the computer. During the closing of the microvalve, the voltage across the corresponding external resistor gradually drops from its maximum value to 0. The closing characteristics of the microvalve, as well as its opening characteristics and the opening and closing characteristics under different pressures, can be analyzed using the data obtained through this process.

### 3.3. Microvalve Characteristic Testing Experiment

**Figure 7:**
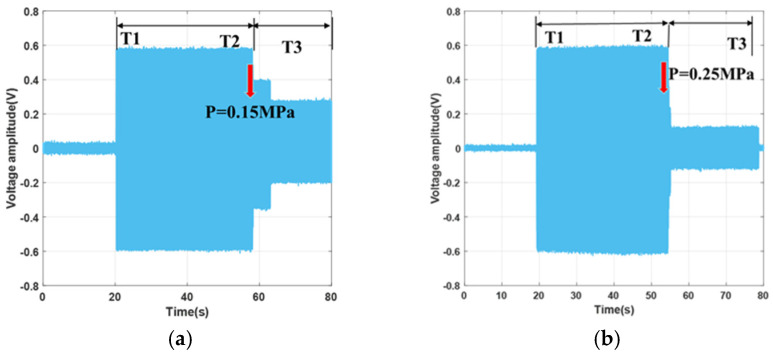
Microvalve closure characteristics under different pneumatic pressures: (**a**) 0.15 MPa; (**b**) 0.25 MPa.

Taking Valve 1 as an example, at the start, all microvalves are in the open state, and the electrolyte solution is injected from Inlet A, flowing through the channel where Valve 1 is located, and then exiting from Outlet E. Subsequently, pressures of 0.15 MPa, 0.25 MPa, and 0.3 MPa are applied to the microvalve. Using the DAQ supervisory control and data acquisition interface, one can observe that the voltage value in Channel 5 sequentially decreases until it reaches 0 at a pressure of 0.3 MPa. Simultaneously, the voltage changes across the external resistor RE, as depicted in Figure 7, showing high-frequency square wave amplitude values displayed as rectangular shapes. The size of the rectangle directly reflects the voltage changes across RE. As indicated in the figures, the electrolyte solution flows from the outlet at time T1, indicating an open flow path; at time T2, pressure is applied, closing the microvalve, which continues until time T3. Minor voltage amplitudes occur both when the flow path is not open and when the microvalve is completely shut off due to power frequency interference. The equivalent resistance of the solution in the microchannel and the sampling resistance are both on the megaohm scale, with a high output impedance, making it susceptible to inductive coupling noise.

According to the experimental results, when P = 0.3 MPa, the microvalve is fully shut off, consistent with observations shown on the display interface. This value is higher than the 0.25 MPa predicted in the simulation results. The reasons for this discrepancy are as follows: (1) fabrication errors, since the actual height of the microchannel is 114.2 μm, which is greater than the ideal value of 100 μm; (2) the actual thickness of the microvalve membrane is 107.3 μm, which is also greater than the ideal value of 100 μm; and (3) misalignment during bonding, resulting in the microchannel not being perfectly centered under the microvalve.

**Figure 8:**
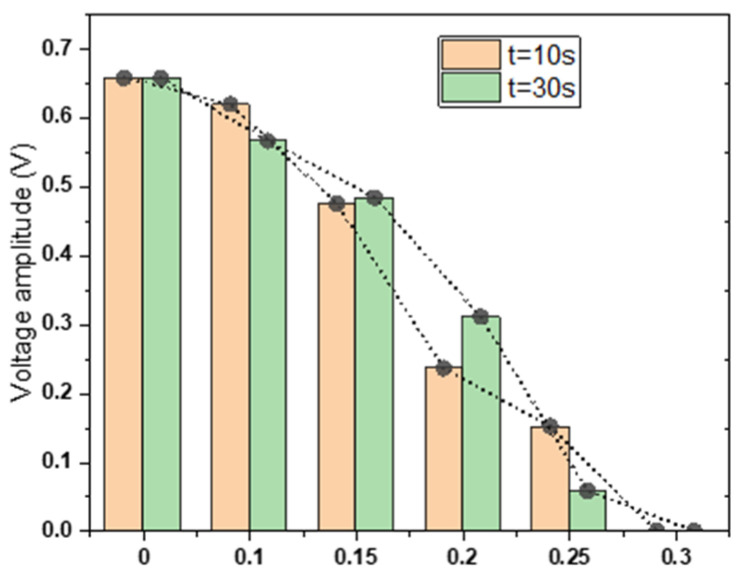
Voltage amplitude measurements at 10 s and 30 s under varying pneumatic pressures.

Further analysis, comparing the changes in voltage across the external resistor after the valve was closed for 10 s and 30 s, reveals significant insights. From the results in Figure 8, it is evident that at P = 0.25 MPa, the deformation of the microvalve shows a substantial difference between 10 s and 30 s. This observation indicates that the greater the applied pressure, the larger the deformation of the microvalve; each microvalve has its optimal closing pressure, at which it closes swiftly. Moreover, as the pressure approaches this optimal value, the longer the pressure is applied, the greater the deformation of the microvalve.

**Figure 9:**
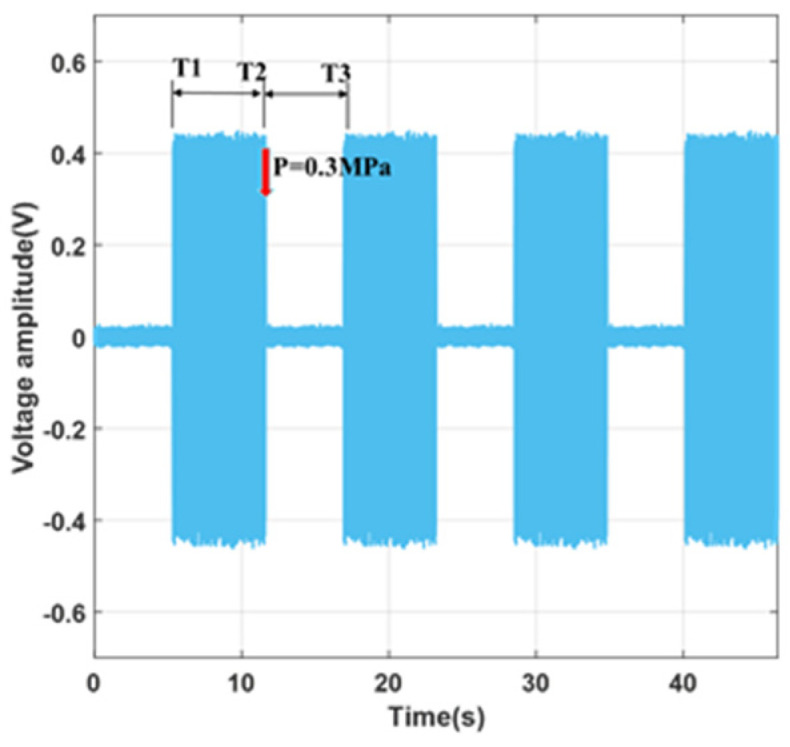
Experimental results of the continuous opening and closing of microvalves at 0.3 MPa.

To verify the reproducibility of the microvalve’s opening and closing characteristics, continuous repetition of opening and closing operations was performed on Valve 1 at P = 0.3 MPa, with the results shown in the graph below. At the moment T1, the solution conducts; at the moment T2, pressure is applied, closing the microvalve; at the moment T3, the microvalve is reopened. The process is repeated twice thereafter. The results of Figure 9 indicate that the microvalve’s opening and closing characteristics are highly repeatable. To further observe the dynamic characteristics of the microvalve closing process, details of the microvalve closing were magnified, showing that the microvalve fully closes within a time t < 1.5 s, indicating a fast closure response.

This demonstration of reproducibility and quick valve response is crucial for applications requiring precise and reliable control over fluid flow, such as in automated microfluidic systems for chemical or biological assays. The ability to rapidly and consistently open and close the valves provides significant advantages in terms of controlling reaction times, sample volumes, and flow rates, making such microvalves invaluable components in the design of efficient and scalable microfluidic devices.

## 4. Microfluidic System Application in SPR Array Sensing

**Figure 10:**
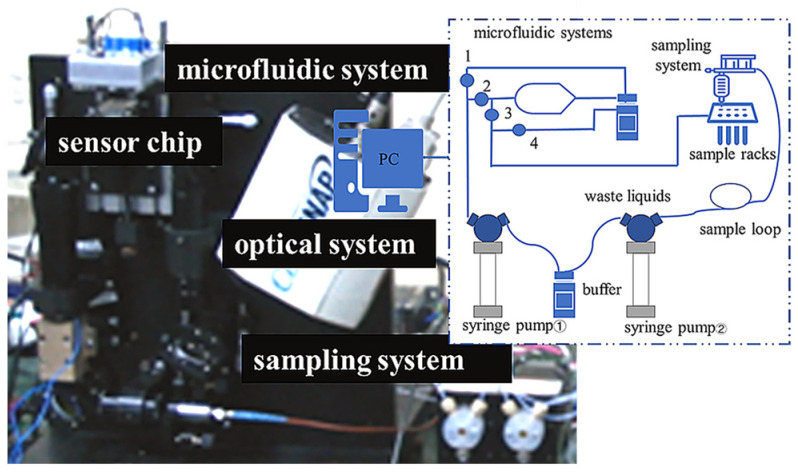
Photograph of microfluidic system fixed on the SPR array system.

An experiment was conducted on a dual-channel differential interferometric imaging SPR array detection device constructed by our research group. The microfluidic system equipped with microvalves was mounted on the prism holder of the device via screws, as shown in Figure 10.

### 4.1. Pre-Experiment for Cleaning the Tubing

**Figure 11:**
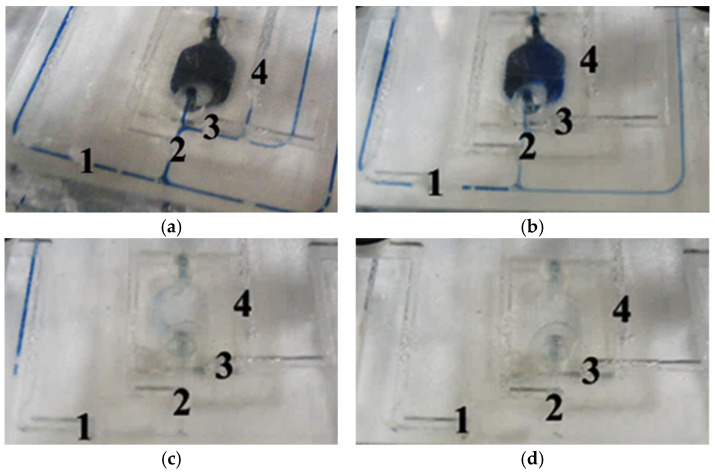
Observations of changes during cleaning experiments: (**a**) initial state; (**b**) opening of the pneumatic Microvalves 2 and 4; (**c**) instant change in microfluidic fouling; (**d**) final microfluidic state.

The pipeline cleaning experiment commenced by manually introducing iron gallic ink (which mainly consists of ferrous sulfate, tannic acid, water, phenol, and gum arabic) into the tubing and onto the microfluidic chip to introduce contaminants in the system. Subsequently, an automated command sequence for cleaning the pipelines was executed to observe the changes in the contamination present on the microfluidic chip. The initial state of the contamination on the microfluidic chip is shown in Figure 11a, while Figure 11b depicts the contamination during the simultaneous closure of Microvalves 2 and 4 with the injection of a buffer solution, revealing that the tubing at Valve 4 has been cleaned and the concentration of contamination in the reaction pool has also reduced. Figure 11c represents the state of microfluidic contamination at the moment of switching, and Figure 11d shows the state of the microfluidic system after the cleaning process has concluded. The entire experiment was carried out automatically through the cleaning command sequence, demonstrating that the microvalves meet the experimental requirements. This automated cleaning process highlights the effectiveness of using an integrated control system for the microvalves and the microfluidic chip to manage and reduce contamination within the system.

### 4.2. SPR Array Detection Experiments

#### 4.2.1. Automatic Sampling Experiments and Results

SPR was used to detect changes in the refractive index of a gold film surface, whereby solutions of different refractive indices flow across the surface of the gold film and generate a corresponding signal.

A 5% NaCl solution was selected as the sample solution for the experiment because the positive correlation between the refractive index of the solution and its concentration provides insightful information on the functionality and precision of the microvalves in the proposed SPR array detection system. The experiment’s setup, which uses deionized water as the buffer and a flow rate of 100 μL/min, outlines a clear method for evaluating the baseline status and responses to the NaCl solution introduction. The experimental procedure is structured as follows to ensure the accuracy of the SPR measurements:

(1) Deionized water is injected for 60 s to establish the baseline. This step is crucial for SPR experiments as it provides a reference point against which changes in the refractive index can be measured;
(2) The microvalve is opened to introduce the NaCl solution for 240 s. This longer duration ensures that the NaCl solution has sufficient time to interact with the sensing area, facilitating stable refractive index measurements;
(3) Deionized water is reintroduced to flush the system and conclude the experiment. This step helps to return the system to its initial state and verifies the reversibility of the interaction on the sensing surface.

**Figure 12:**
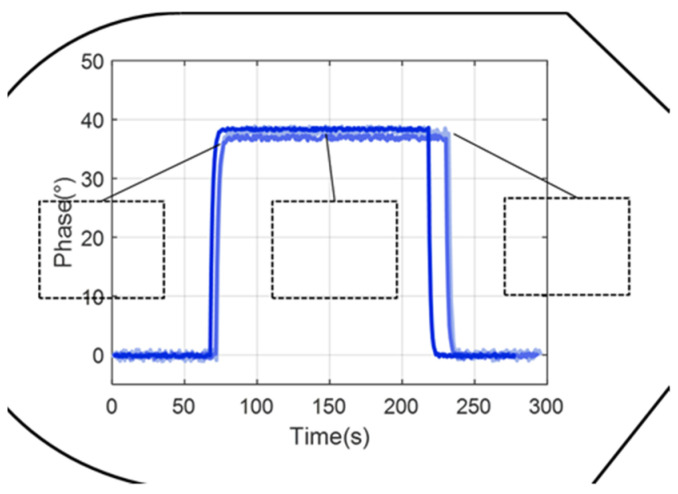
Detection results in different regions.

To analyze the consistency of the detection results, three 10 × 10-pixel sections (where the pixel size is 6.45 μm × 6.45 μm) within the detection zone of the sensing surface from the inlet to the outlet of the microfluidic pool were selected. And calculating the phase angle changes at these positions, is a rigorous approach, as shown in Figure 12. The nearly identical phase angle changes obtained from the microvalve switching demonstrate the microvalves’ effectiveness at automating sample introduction, thereby meeting the requirements for SPR array detection. This uniformity in phase change across different areas indicates a consistent sample delivery and interaction across the sensing surface, which is essential for reliable SPR measurements.

#### 4.2.2. Analysis and Results of Microvalve Repeatability Experiments

**Figure 13:**
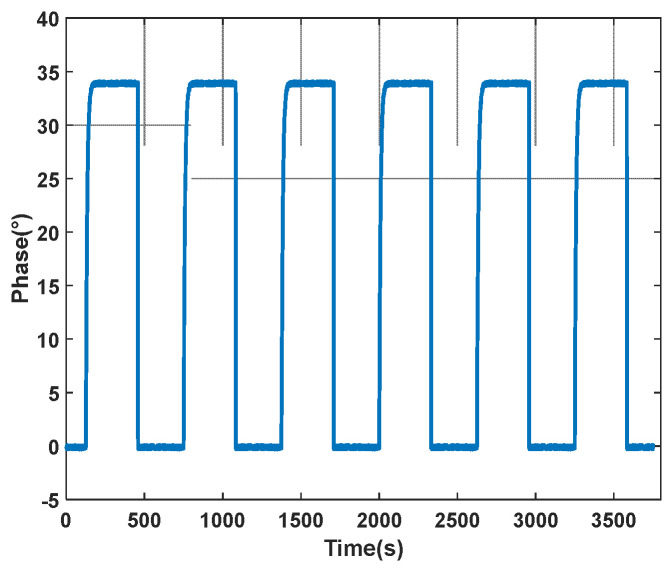
Experimental outcomes from the repeated activation of microvalves.

Biological experiments often require frequent switching of the microvalves within a matter of minutes to change the solutions entering the microfluidic pool. Thus, the repeatability of the microvalves’ operation is a critical factor in ensuring the accuracy and comparability of experimental results. On a dual-channel differential interferometric imaging SPR detection device, the repeatability of the microvalves was tested by repeatedly measuring changes in the refractive index of the solutions. In the experiment, the flow rate was set to 100 μL/min, with the microvalves switching every 5 min to alternately inject deionized water and 5% NaCl solution into the microfluidic pool. Within the detection area on the sensing surface, a 10 × 10-pixel region was selected for calculation. The results showed that the amplitude’s standard deviation was 0.0013, with the standard deviation of the rise and fall time being 1.3 s (Figure 13). These results confirm the high repeatability of the microvalve switching process in the experimental setup, with three times shorter compared to the four-way valve.

The comprehensive results of the experiments indicate that the designed microvalves, which control the alternating injection of brine at various concentrations and deionized water into the microfluidic chamber, lead to better consistency in the experimental outcomes, thus achieving automated sampling. Furthermore, the control provided by the microvalves significantly enhances both the response time and the rising and falling rates of the SPR curves, while also ensuring good reproducibility.

## 5. Conclusions

This paper describes a microfluidic system equipped with integrated microvalves tailored for SPR array sensing. It explores the dynamic behavior of both individual and collective microvalves through conductance measurement. In the experimental phase, the system was utilized to manage the flow of NaCl solutions via microvalve actuation to facilitate SPR array sensing. The experimental results show the following:

(1) The closing status of the microvalve can be influenced by the control pressure; the microvalve can achieve complete closure at 0.3 MPa with reliable reproducibility;
(2) The microvalve detection method is capable of characterizing both individual and multiple microvalves simultaneously and demonstrating effective parallel detection;
(3) In SPR array sensing tasks, microvalves enable the automation of flow direction and injection processes;
(4) This detection method is versatile, suitable for a range of microfluidic systems equipped with integrated microvalves.

The research establishes the crucial role of microvalves in improving both the functionality and efficiency of microfluidic systems, particularly for precise fluid control in SPR array sensing. Their integration supports automated, accurate experiments, which is essential progress in areas such as biochemistry and pharmacology. This highlights the wide-ranging applicability of microvalves in both scientific research and industrial processes.

## Figures and Tables

**Table 1:** Cross-sectional area of various microchannels, maximum pressure required at cutoff, and maximum shear stress at the connection.

Cross-Sectional Shape	NO.1	NO.2	NO.3	NO.4
Cross-sectional area (mm^2^)	0.0157	0.0344	0.0217	0.0257
Maximum required pressure (MPa)	0.35	0.2	0.25	0.42
Maximum shear stress at connection (MPa)	0.46	0.23	0.32	0.48

## Data Availability

No new data were created or analyzed in this study. Data sharing is not applicable to this article.
